# Decoding onset and direction of movements using Electrocorticographic (ECoG) signals in humans

**DOI:** 10.3389/fneng.2012.00015

**Published:** 2012-08-08

**Authors:** Zuoguan Wang, Aysegul Gunduz, Peter Brunner, Anthony L. Ritaccio, Qiang Ji, Gerwin Schalk

**Affiliations:** ^1^Department of ECSE, Rensselaer Polytechnic Institute, TroyNY, USA; ^2^J Crayton Pruitt Family Department of Biomed Engineering, University of Florida, GainesvilleFL, USA; ^3^BCI R&D Program, Wadsworth Center, New York State Department of Health, AlbanyNY, USA; ^4^Department of Neurology, Albany Medical College, AlbanyNY, USA

**Keywords:** brain computer interface, ECoG, movement direction prediction, movement onset prediction, neurorehabilitatation, performance augmentation

## Abstract

Communication of intent usually requires motor function. This requirement can be limiting when a person is engaged in a task, or prohibitive for some people suffering from neuromuscular disorders. Determining a person's intent, e.g., where and when to move, from brain signals rather than from muscles would have important applications in clinical or other domains. For example, detection of the onset and direction of intended movements may provide the basis for restoration of simple grasping function in people with chronic stroke, or could be used to optimize a user's interaction with the surrounding environment. Detecting the onset and direction of actual movements are a first step in this direction. In this study, we demonstrate that we can detect the onset of intended movements and their direction using electrocorticographic (ECoG) signals recorded from the surface of the cortex in humans. We also demonstrate in a simulation that the information encoded in ECoG about these movements may improve performance in a targeting task. In summary, the results in this paper suggest that detection of intended movement is possible, and may serve useful functions.

## 1. Introduction

Brain-computer interfaces (BCIs) aim to translate a person's intentions into meaningful computer commands using brain activity alone (Wolpaw et al., 2002; Mak and Wolpaw, 2009). In particular, determining when and where a person intends to move would have important clinical applications for those suffering from neuromuscular disorders (Sejnowski et al., 2007; Tan and Nijholt, 2010). For example, a BCI that detects intended movement onset in absence of actual movements could restore grasp function in people with chronic stroke (Buch et al., 2008; Daly and Wolpaw, 2008; Wisneski et al., 2008; Muralidharan et al., 2011; Yanagisawa et al., 2011). Also, a BCI that predicts intended movement onset prior to actual movements would have many practical applications in everyday life. For example, it may support faster braking during vehicle operation (Haufe et al., 2011) or more rapid targeting in military applications (Gunduz and Schalk, 2011).

The first step in this direction is to establish whether it is possible to detect the onset of actual movements from brain signals. Several previous studies have shown that intracortical activities recorded in primates over the premotor or parietal cortices are related to the onset of movements (Achtman et al., 2007; Lebedev et al., 2008; Hwang and Andersen, 2009; Hasan and Gan, 2011; Mirabella et al., 2011), but access to intracortical activity in humans has been scarce (e.g., Hochberg et al., 2006; Simeral et al., 2011). Other studies have investigated movement onset using electroencephalographic (EEG) signals in humans (Mason and Birch, 2000; Millan and Mouriño, 2003; Borisoff et al., 2004; Leeb et al., 2007; Bai et al., 2008; Hasan and Gan, 2011; Muralidharan et al., 2011), but accurately detecting the corresponding EEG signatures in single trials has proven difficult. Electrocorticographic (ECoG) signals are recorded directly from the surface of the cortex, and thus have a higher signal-to-noise ratio compared to EEG (Ball et al., 2009). They also readily support detection of certain physiological phenomena, such as high gamma activity (>70 Hz), that is largely inconspicuous on the scalp. The ability to detect high gamma activity is an important advantage, since many ECoG studies (e.g., Miller et al., 2007, 2009; Kubánek et al., 2009; Chao et al., 2010) demonstrated that spatially focused high gamma activity correlates closely with specific aspects of motor functions. Yet, no previous study comprehensively studied the possibility that movement onset can be detected using ECoG signals.

The second step in this direction is to determine whether ECoG also holds information about movement direction prior to the actual movement. Several studies (Schalk et al., 2007; Pistohl et al., 2008; Gunduz et al., 2009) showed that ECoG signals collected during movements hold information about two-dimensional trajectories of hand movements, and Leuthardt et al. (2004) and Schalk et al. (2008) demonstrated one- and two-dimensional real-time control of a computer cursor using ECoG, respectively. However, there has only been scarce evidence that brain signals recorded in humans give information about movement direction prior to the movement (Leuthardt et al. (2004) using ECoG, Wang et al. (2010) using MEG), and that this information may be useful.

In this paper, we investigate whether ECoG holds information about the onset and direction of hand movements in a center-out task. Specifically, we use a support vector machine (SVM) classifier to determine, at each step in time, the probability that the subject initiated a hand movement at that particular time. We also characterize the ECoG features that hold the most information about movement onset. We then use a novel implementation of a time-varying dynamic Bayesian network (TVDBN), which was designed to take advantage of the spatio-temporal dynamics of ECoG features, to determine the direction of the intended movement using ECoG signals prior to the movement. Finally, we simulate a targeting application in which brain signals prior to the movement are combined with the actual movement signals. In this simulation, the time-to-target reduces by up to 150 ms when we use the directional information captured in ECoG signals. Overall, our results contribute to our understanding of the neural representation of intended movements and suggest that integrating information from brain signals and motor execution may eventually lead to systems that can improve a user's performance.

## 2. Materials

### 2.1. Human subjects

**Figure A1:**
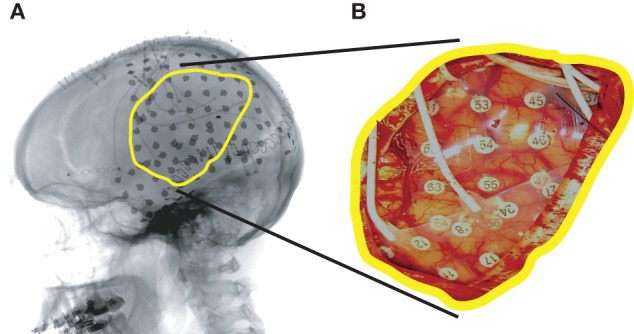
**Example of an implanted subdural grid (Subject A). (A)** Lateral radiograph indicating grid position. **(B)** Subdural grid placed over left fronto-parietal and temporal lobes.

Five subjects participated in this study. The subjects were patients with intractable epilepsy who underwent temporary implantation of subdural electrode arrays for the localization of seizure foci prior to surgical resection. Table 1 summarizes the subjects' clinical profiles. All of the subjects had normal cognitive capacity and were functionally independent. The study was approved by the Institutional Review Board of Albany Medical College as well as by the Human Research Protections Office of the US Army Medical Research and Materiel Command, and the subjects gave informed consent. The implanted electrode grids (Ad-Tech Medical Corp., Racine, WI) consisted of platinum-iridium electrodes that were 4 mm in diameter (2.3 mm exposed) and were configured with an inter-electrode distance of 1 cm. Subject E was implanted with a higher density (6 mm inter-electrode distance) grid with 68 contacts (PMT Corp., Chanhassen, MN) over the temporal lobe. Each subject had postoperative anterior-posterior and lateral radiographs (see Figure A1), as well as computer tomography (CT) scans to verify grid location. The number of implanted electrodes varied between 58 and 120 contacts across subjects (Table 1). We excluded data collected over the occipital strips (in Subjects A and B) from the analyses to minimize the potential impact of visual stimulation on the results.

**Table 1:** **Clinical profiles of the subjects that participated in the study**.

**Subject**	**Age**	**Sex**	**Handedness**	**Perf. IQ**	**Seizure focus**	**Grid/Strip location**	**# of Electrodes**
A	29	F	R	136	Left temporal	Left fronto-parietal	64
						Left temporal	23
						Left temporal pole	3
						Left occipital	6
B	56	M	R	87	Left temporal	Left frontal	56
						Left temporal	35
						Left occipital	6
						Right posterior mesial	4
C	45	M	R	95	Left temporal	Left fronto-temporal	54
						Left temporal pole	4
D	49	F	L	99	Left temporal	Left fronto-temporal	61
						Left temporal mesial	4
						Left frontal	4
E	29	F	R	95	Left temporal	Left frontal	40
						Left temporal	68
						Left frontal	4
						Left inferior temporal	4
						Left parietal	4

### 2.2. Cortical mapping

**Figure 1:**
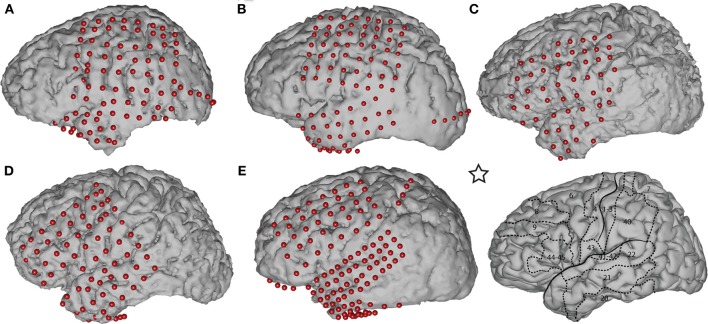
**Subject-specific brain models and projected electrode locations for Subjects A–E**. The brain template on the bottom right, indicated with a star, depicts the location of the central sulcus, Sylvian fissure, and important Brodmann areas. The areas most relevant to our task are Brodmann areas 6 (premotor), 4 (primary motor), 1–3 (sensory motor), and 7 (posterior parietal cortex).

We used Curry software (Compumedics, Charlotte, NC) to create subject-specific 3D cortical brain models from high resolution pre-op MRI scans. We co-registered the MRIs with post-op CTs and extracted the stereotactic coordinates of each grid electrode. We identified the cortical areas underneath each electrode using an automated Talairach Atlas (Lancaster et al., 2000) (http://www.talairach.org/daemon.html) for functional mapping. We also projected the electrodes onto the reconstructed brain models (see Figure 1) and generated activation maps using custom Matlab software to delimit the cortical areas involved in prediction of movement onset and direction.

### 2.3. Data collection

We recorded ECoG signals at the bedside using eight 16-channel g.USBamp biosignal acquisition devices (g.tec, Graz, Austria) at a sampling rate of 1200 Hz. Electrode contacts distant from epileptic foci and areas of interest were used for reference and ground. In addition to recording brain activity, we also recorded the subjects' eye gaze using a monitor with a built-in eye tracking system (Tobii Tech., Stockholm, Sweden) positioned 54–60 cm in front of the subjects, and the movements of a joystick. The eye tracker was calibrated to each subject at the beginning of the experimental session using custom software that invoked standard calibration functions provided by Tobii. Data collection from the biosignal and behavioral acquisition devices (g.USBamp, eye tracker, and joystick, respectively), as well as control of the experimental paradigm and stimulus presentation, were accomplished simultaneously using BCI2000 software (Schalk et al., 2004; Schalk and Mellinger, 2010). BCI2000 provides a flexible general-purpose software platform that consists of modules that realize signal acquisition, signal processing, user feedback, and an operating protocol. BCI2000 facilitates the implementation of any BCI or related system, and is used in hundreds of laboratories for this purpose.

### 2.4. Experimental paradigm

**Figure 2:**
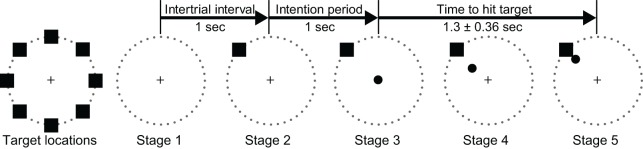
**Illustration of all eight possible target locations (left) and the five experimental stages**.

**Figure A2:**
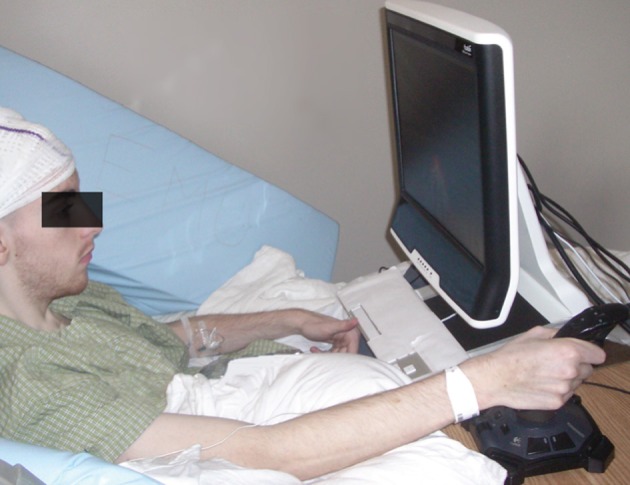
**The experimental setup at Albany Medical College**. Joystick control mostly entailed wrist movements.

ECoG signals were collected while the subjects performed an 8-target center-out cursor movement task (Georgopoulos et al., 1982) while fixating their eye gaze at a central fixation cross. Eye gaze fixation was enforced online by BCI2000: a trial was aborted if the subject looked away from the center for more than 5° for more than 500 msec. Each trial started with the presentation of a target in one of eight possible locations. A cursor appeared 1 s later at the center of the screen. The subjects' task was to use their hand contralateral to the implant(s) to control a joystick so as to move the cursor into the target. (Only Subject D used the non-dominant hand.) We positioned the subjects such that the joystick movements were mainly restricted to the wrist (see Figure A2). The subjects were instructed to make exaggerated movements and achieve maximal radial extension of the joystick to hit the targets. Once the target was hit, the next trial started after an inter-trial interval of 1 s. Figure 2 gives a simple illustration of the stages of the task. Trials aborted by the eye tracker, trials in which joystick movement preceded the presentation of the cursor, and trials in which subjects failed to hit the correct target were omitted from further analyses. The total number of remaining valid trials were 394, 584, 258, 398, and 305 for Subjects A through E, respectively.

## 3. Methods

The primary goal of this study was to determine whether ECoG may be used to detect the onset and direction of an intended movement, and whether this information could be useful to reduce the time-to-target in a simulated targeting application. In the following sections, we describe our methods for ECoG feature extraction, movement onset and direction prediction, and the simulation of the targeting application.

### 3.1. Feature extraction

**Figure 3:**
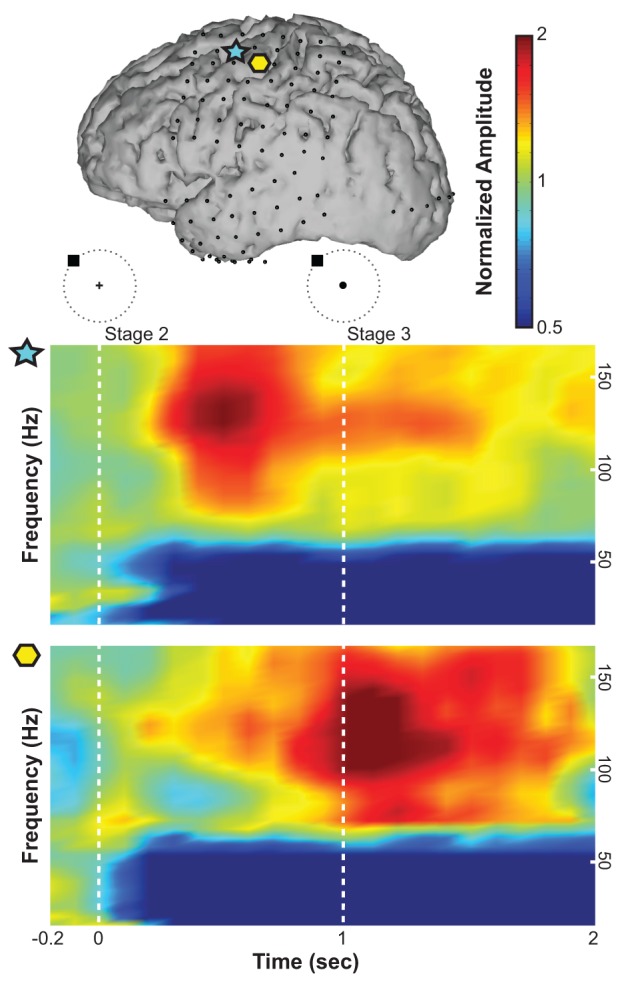
**Exemplary time-frequency plots from Subject B of normalized spectral amplitude (indicated with a linear colorscale) for an electrode over the premotor cortex (indicated by the blue star in the brain model on top) and an electrode over primary motor cortex (yellow hexagon)**. The horizontal axis gives time (the target was presented at time 0; the cursor was presented at 1 s). The vertical axis gives frequency.

We first re-referenced the raw ECoG signals (excluding occipital channels) using a common average reference (CAR) spatial filter to remove spatial noise (Kubánek et al., 2009). For each 100 ms time step and each channel, we converted 300 ms windows (i.e., 200 ms overlap) of ECoG time series into the frequency domain using an autoregressive model of order 25 (Marple, 1986). Using this model, we derived frequency amplitudes between 0 and 200 Hz in 1 Hz bins. Figure 3 shows an example of ECoG activity at different frequencies during the preparation for and execution of the movement task with respect to rest averaged across trials. Spectral amplitudes were divided by an average spectrum of the rest condition. A normalized amplitude of 1 suggests no task-related modulations at a particular time and frequency, whereas a value of 2, for instance, suggests that the spectral amplitude of interest doubled during the task. ECoG features were attained by averaging these frequency amplitudes across three frequency bands: mu (8–12 Hz), beta (18–26 Hz), and high gamma activity (70–170 Hz). In addition to these three spectral features, we also calculated the local motor potential (LMP) (Schalk et al., 2007; Kubánek et al., 2009) by averaging the raw time-domain signal at each channel over each 300 ms time window (also 200 ms overlap). This process resulted in a total of four features from each ECoG channel at each (100 ms) time step.

To remove features unrelated to movement onset and direction prediction, we performed feature selection via forward search on five cross-validation folds (i.e., dividing the number of total trials into five and using four-folds for training and one-fold as the novel testing set, five times). The algorithm started with an empty feature set and, at every iteration, added a new feature to the set to generate best classification accuracy across each of the five testing folds. We chose the size of features as 20 for both movement onset prediction and movement direction prediction.

### 3.2. Prediction of movement onset

We downsampled the joystick data to 10 Hz using a moving average filter (300 ms window, 200 ms overlap) to align with the ECoG features. In each trial, we defined the actual movement onset as the time sample when the joystick was pushed beyond one eight of its maximum radial extension from its rest position. For each trial, there was a single onset sample and all time samples from the beginning of the trial up to this onset were labeled as not onset.

We then designed a detector that accumulated 1 s of ECoG features into a first-in-first-out (FIFO) buffer and determined from a full buffer whether the subsequent time step would be the onset of a movement. Each trial started with an empty buffer which was updated with new features every 100 ms. Once the buffer was full, a prediction was made every 100 ms via a weighted SVM (Huang and Du, 2005). We opted for a weighted SVM as it overcomes the classification bias that results from the unbalanced nature of the data (i.e., the class not onset' is much more likely than the class onset) by setting the ratio of penalties to the inverse ratio of the class sizes. We configured the weighted SVM to use a radial basis function as the kernel. The labeled joystick data was divided into 5-folds; four of these folds were used for training the weighted SVM, and one fold was allocated for testing. We repeated this process five times until each fold was used for testing^1^.

The output of the weighted SVM classifier yielded the probability of an onset as a function of time. Time points were classified as movement onsets if their probability values were greater than an empirically determined threshold of 0.3. We chose the *F*1-score as our accuracy metric as it is preferable over percent accuracy or error rate for highly unbalanced classes (van Rijsbergen, 1979). *F*1-score is defined as:

(1) $$F1=\frac{2\text{TP}}{2\text{TP}+\text{FP}+\text{FN}}$$

where TP, FP, and FN are the occurrences of true positive, false positive, and false negative predictions. *F*1-score is particularly befitting for our onset predictor, as the metric is not influenced by true negative (TN) predictions. Given the imbalance of our two classes, any random classifier is likely to yield high TN rates. *F*1-scores take on values between 0 and 1, with the latter corresponding to a perfect classifier.

We computed the *F*1-scores of onset predictions for all subjects. We were also interested in determining the ECoG features (i.e., spectral bands and spatial locations) that were most predictive of movement onset. To do this, we calculated *F*1-scores separately for each ECoG feature using the methods described above. We ran the 5-fold cross validation 20 times; each time there was a random division of the folds. A series of high *F*1-scores suggests that the prediction was highly accurate, whereas ratios close to zero indicate a poor predictor. Hence, we tested whether the *F*1-scores in these 20-folds were significantly different than zero via a *t*-test. The corresponding *p*-values represent the significance of the *F*1-scores, and thus the accuracy of the classifier. We converted these *p*-values into indices of confidence [(i.e., −*log*(*p*))], and mapped those confidence indices on the cortex models of the individual subjects.

### 3.3. Prediction of direction of intended movement

We predicted movement direction using all ECoG features from 1s prior to the actual movement onset (i.e., the same window used to predict the movement onset). Routinely in such multivariate prediction problems, multichannel time series are re-arranged into a vector to be used as input features to train a classifier, e.g., a neural network or SVM. However, the shortcoming of this approach is that it generally ignores the spatial and temporal structure of the multidimensional time series. Because we felt that such structure was likely important for directional classification, we opted to implement a novel modified time-varying dynamic Bayesian network (MTVDBN) that can capture the spatial and temporal dependency of the ECoG signals across both domains.

**Figure 4:**
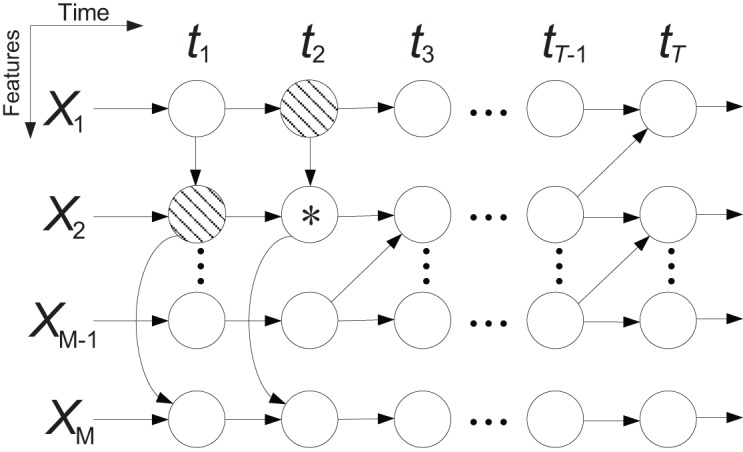
**Block diagram of a modified time-varying dynamic Bayesian network (MTVDBN)**. The shaded nodes are the parents of the node *.

A Bayesian network is a probabilistic graphical model that represents a set of variables and their conditional dependencies via directed acyclic graphs, in which nodes represent random variables and edges represent conditional dependencies. A Bayesian network is an established method for modeling dependency structures in complex multivariate systems. An extension of Bayesian networks that models time series is called a dynamic Bayesian network (DBN). However, an important assumption underlying DBNs is that the time series are generated by a stationary process, which generally does not hold for neural signals. TVDBN (Song et al., 2009) introduced non-stationary temporal transitions, but did not describe spatial dependency between variables. In this paper, we extend the TVDBN to the modified TVDBN (MTVDBN) in which the non-stationary temporal and spatial dependencies of ECoG signals are modeled simultaneously. Figure 4 depicts a block diagram of a MTVDBN in which (vertical) arrows within each time slice (i.e., across rows of a column) represent spatial dependencies, while (horizontal) arrows across time slices (i.e., across columns) describe the temporal dependencies. Regular TVDBNs lack the former spatial structure, i.e., the (vertical) arrows within each time slice.

Let **X**^*t*^ = (*X*^*t*^_1_, …, *X*^*t*^_M_)^*T*^ be a vector representing the ECoG features (mu, beta, high gamma bands, and LMP) from all channels at time *t* (i.e., *M* is four times the number of channels for each subject). The joint likelihood of the time sequence of length *T* can be expressed as:

(2) $$P(X^{1},...,X^{T})=\prod_{t=1}^{T}\prod_{m=1}^{M}P(X_{m}^{t}|X_{\pi_{m}}^{t-1},\;X_{\pi_{m}}^{t}),$$

where **X**^*t*−1^_π_*m*__ and **X**^*t*^_π_*m*__ denote the parents of input feature *X*^*t*^_*m*_ at time (*t*−1) and *t*, respectively (see Figure 4). Since we use 1 s of ECoG features, we are interested in a time sequence of length *T* = 10 samples. Note that the parent **X**^*t*−1^_π_*m*__ represents temporal dependencies, where as parent **X**^*t*^_π_*m*__ represents spatial dependencies. An equivalent form of representing *P*(*X*^*t*^_*m*_|**X**^*t*−1^_π_*m*__, **X**^*t*^_π_*m*__) is the following linear model (Duda et al., 2008):

(3) $$X_{m}^{t}=a_{m}^{t-1}X_{\pi_{m}}^{t-1}+a_{m}^{t}X_{\pi_{m}}^{t}+\;\varepsilon ,\text{ where }\varepsilon \sim N(0,\;1),$$

**a**^*t*−1^_*m*_ and **a**^*t*^_*m*_ are row vectors of the coefficients of parents of feature *X*^*t*^_*m*_ at times (*t*−1) and *t*, respectively, and the variable ε is Gaussian noise with mean zero and unit standard deviation. The coefficients **a** represent the structure of the network. The zero elements of **a** represent the missing links within the structure, whereas the non-zero elements stand for the dependence strength. The coefficients are learned through maximizing the likelihood of *X*^*t*^_*m*_ across all training samples. To prevent overfitting and to encourage sparse structures, we learn **a** through an ℓ_1_ penalty. Specifically, the coefficients **a**^*t*−1^_*m*_ and **a**^*t*^_*m*_ are learned by:

(4) $$\underset{a_{m}^{t-1},\;a_{m}^{t}}{\min}\frac{1}{N}\sum_{n=1}^{N}{\left(X_{m}^{n,\;t}-a_{m}^{t-1}X_{\pi_{m}}^{n,\;t-1}-a_{m}^{t}X_{\pi_{m}}^{n,\;t}\right)}^{2}+\lambda (||a_{m}^{t-1}|{|}_{1}+||a_{m}^{t}|{|}_{1}),$$

where *N* is the size of training vector samples, *X*^*n,t*^_*m*_ represents the data at the feature *m* at time *t* in the *n*^*th*^ training vector. Similarly, **X**^*n,t*^_π_*m*__ is the parent of *X*^*n,t*^_*m*_ at time *t* in the *n*^*th*^ training sample. Parameter λ is the penalty coefficient, which controls the sparsity of the structure and it is identified by cross validation. Equation (4) is solved by least angle regression and shrinkage (LARS) (Efron et al., 2004), which has a computational complexity of *O*(*N*(2*M*−1)^3^) time. Equation (4) was applied to each node to select the potential parents. However, this preprocessing does not automatically result in acyclic graphs at each time slice. A hill-climbing algorithm was then used to greedily construct an acyclic graph in which the edges were restricted by the selected potential parents (Schmidt et al., 2007).

We built a MTVDBN for each direction trained using only the trials toward that direction. For each direction *d* = {1, 2, …, 8}, the set of parameters **a**^*t*^_*d,m*_ for *m* = {1, 2, …, *M*} and *t* = {1, 2, …, *T*} were trained. Each MTVDBN thus learned the patterns of the input ECoG features associated with planning a movement in their assigned direction (Equation 3). After these model parameters were trained, given a test sample of ECoG features, the classification was done by:

(5) $$\begin{array}{l} d^{*}=\underset{d}{\arg\min}\sum_{t=1}^{T}\sum_{m=1}^{M}-\log P(X_{m}^{*,\;t}|X_{\pi_{m}}^{*,\;t-1},\;X_{\pi_{m}}^{*,\;t})\\ \;\;\;\;\;=\underset{d}{\arg\min}\sum_{t=1}^{T}\sum_{m=1}^{M}{\left(X_{m}^{*,\;t}-a_{d,\;m}^{t-1}X_{\pi_{m}}^{*,\;t-1}-a_{d,\;m}^{t}X_{\pi_{m}}^{*,\;t}\right)}^{2}. \end{array}$$

In other words, every time an onset was detected, 1 s of input features (i.e., *T* = 10 samples) were fed into eight MTVDBNs. Each of these eight MTVDBNs yielded the joint likelihood of X^*^ for one particular direction, *d*. In each trial, we then chose the direction that yielded the highest likelihood (i.e., lowest negative log-likelihood) as the predicted direction.

### 3.4. Integration of ECoG-based predictions and task

**Figure 5:**
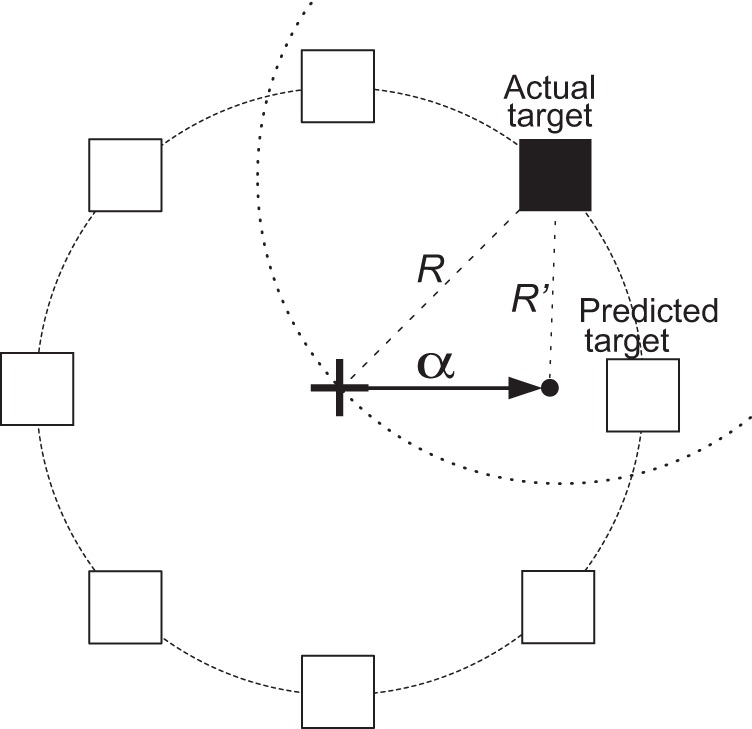
**An illustration of the simulated brain-assisted targeting system**. The black box depicts the current target and the white boxes represent all other possible target locations. The arrow shows the incorrectly predicted direction. The dashed circle is the locus of all points at a distance *R* away from the target. When the cursor is re-positioned in this circle, the distance to the target is decreased, as *R*′ < R.

In additional offline analyses, we simulated the integration of the directional prediction using ECoG signals with the task. We placed the cursor in the predicted direction at a distance α from the center. In other words, if the directional prediction was accurate, the cursor would be placed closer to the target, resulting in a decreased time needed to hit the target. Figure 5 gives a simple illustration of re-positioning the cursor toward the predicted target direction. In this figure, the black box is the target and the predicted direction is (somewhat inaccurately) towards the right. If the radius of the circle in Figure 5 is *R*, to decrease movement time, the distance between the re-positioned cursor and the target should be less than *R*. The dashed circle is the locus of all points at a distance *R* to the target. Thus, to be closer to the target, after direction prediction the cursor should lie within the dashed circle. The area enclosed by the two circles is 39% of the area of the solid circle, implying that if we place the cursor in a random direction, then there would be a 61% chance that the cursor would be farther away from the target compared to if were left in the center of the circle. In other words, if we randomly placed the cursor within the solid circle, on average it would take the subject longer to hit the target. Conversely, if the output prediction is the correct target or at least one of its neighbors, this would bring the cursor closer to the target and may allow the user to reduce the time it would take to hit the target. The angle between the imaginary line that passes through the center and the actual target and the imaginary line that passes through the center and one of the intersection points between the two circles is 60°. Hence, we have an error margin of ±60° to improve the performance.

## 4. Results

### 4.1. Prediction of movement onset

**Figure 6:**
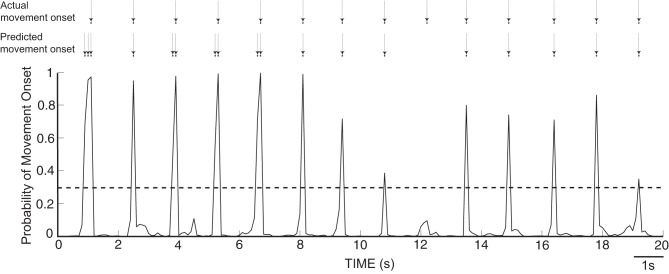
**Time course of likelihood of movement onset for Subject A**. The dashed line represents a probability level of 0.3, which was used as a threshold to predict the movement onset.

As described in the Methods, we computed from ECoG signals (i.e., amplitudes in the mu, beta, and gamma band, as well as the LMP) the likelihood of an occurrence of a movement onset. Figure 6 gives an exemplary resulting time course of the likelihood values of an onset over 20 s for Subject A, along with arrows that indicate the actual and predicted movement onsets. We observe that the likelihood curves exhibit sharp peaks around the actual movement onset, which demonstrates that the classifier can accurately detect movement onsets using ECoG signals. In each subject, we used these likelihood values to classify each time point *t* as “onset” if the likelihood value exceeded an empirically attained threshold value of 0.3. The confusion matrices, evaluated during actual movement, for all five subjects are shown in Table 2 in percentages, along with the total number of events and the resulting *F*1-scores. Most of the false positives occur 200 ms around the movement onset (i.e., the peak of the probability function). The predictor yields high detection performance for Subjects A and B. The high occurrence of false negatives decreased the *F*1-score for Subjects D and E. The high number of false negatives and false positives led to reduced performance in Subject C.

**Table 2:** **Confusion matrices of movement onset prediction for Subjects A–E. The values shown in parentheses are the false positives that occur more than 200 ms away from the actual movement onset. The bottom row gives the *F*1-score**.

	**Subject A**		**Subject B**		**Subject C**		**Subject D**		**Subject E**	
	**Actual not onset**	**Actual onset**	**Actual not onset**	**Actual onset**	**Actual not onset**	**Actual onset**	**Actual not onset**	**Actual onset**	**Actual not onset**	**Actual onset**
Predicted not onset	94%	27%	94%	36%	73%	74%	84%	65%	93%	66%
Predicted onset	6%(0.5%)	73%	6%(0.7%)	64%	27%(22%)	26%	16%(9%)	35%	7%(4%)	34%
Number of events	3991	316	4021	468	4537	205	3380	316	4640	320
*F*1-score	0.69		0.63		0.06		0.23		0.35	

**Figure 7:**
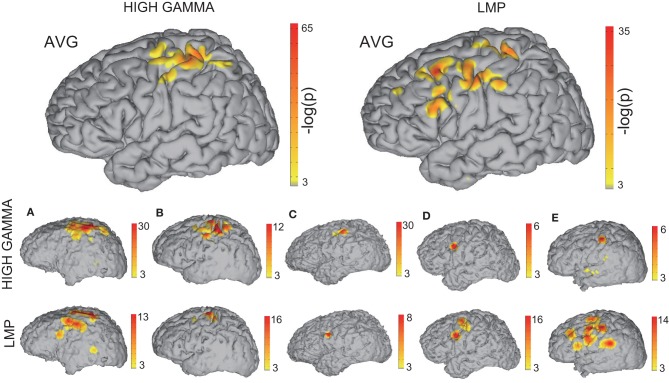
**Relationship of brain signals with movement onset**. This figure shows the spatial distribution of accumulated confidence indices −*log*(*p*) for the 70–170 Hz high gamma band (top left) or the LMP (top right) across all subjects. The spatial distribution of the confidence indices for each subject are depicted in the bottom rows.

Figure 7 shows the confidence indices of the ECoG features that are most predictive of the movement onset. (Note that a significance level of *p* < 0.05 corresponds to a confidence index of −*log*(*p*) > 3). The two larger brain models in the top row show the confidence indices for the high gamma band and LMP, the most predictive of the features, accumulated across all subjects. The bottom two rows show the results of the high gamma band and LMP for individual Subjects A–E. These results indicate that the brain areas yielding prediction of movement onset are centered on hand representations of motor cortical areas for the high gamma band and extend beyond the hand representations for the LMP. This finding is in agreement with results from a previous report that investigated finger flexions (Kubánek et al., 2009). However, areas other than the sensorimotor cortex were used in the onset prediction as selected by the feature selection algorithm (see Methods). On the other hand, mu and beta bands did not yield statistically significant activations for prediction of movement onset. Although desynchronization of these bands has been posited to relate to local increases in high gamma amplitude in the motor cortex (i.e., as a gating mechanism) (Miller et al., 2009), the corresponding brain signals are known to be spatially widespread and slowly evolving, and thus may not be the best indicators for the exact timing of movement onset.

### 4.2. Prediction of direction of intended movement

We computed the absolute value of the angular error of the directional predictions obtained by the MTVDBNs for each trial and each subject, and calculated their mean and standard deviation across all trials. This yielded the following single-trial angular error statistics for Subjects A–E, respectively: 55.29° ± 52.61°, 46.28° ± 57.80°, 68.95° ± 55.16°, 70.55° ± 53.78°, and 87.8° ± 57.9°. A single-sided *t*-test revealed that the accuracy of our results was better than chance (i.e., 90.0 ± 57.36°) at a significance level of 5% (*p* < 0.05) for all subjects. Our method yielded single-trial angular errors that were smaller than 90° for all subjects, and as low as 46° in one subject. Given that the targets are separated by 45°, the results indicate that the classifier was able to infer the direction of intended movements within less than two targets in single trials.

### 4.3. Integration of ECoG-based predictions and task

**Figure 8:**
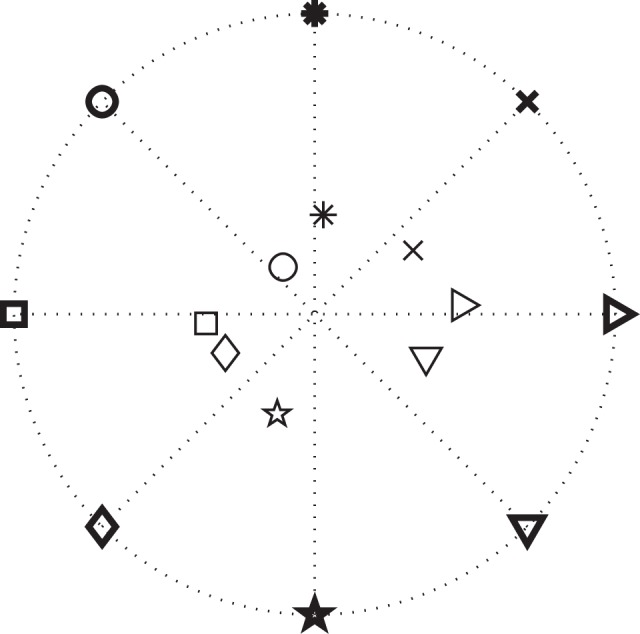
**Results for movement direction prediction results for Subject A**. The symbols represent the eight different targets. The symbols in bold depict the actual positions of the targets and the thinner symbols show the averaged pre-movement positions of the cursor across all trials in each direction. (Note that these are average cursor positions. Values for single-trial angular errors are given in the text.)

We further studied whether integrating information extracted from ECoG signals would improve performance in a simulated targeting task. Every time a movement onset and direction were predicted, we placed the cursor in the predicted direction. As described in the Methods, this stimulation will result in improved performance if the single-trial error of the predicted direction is less than 60°. Figure 8 shows the positions of the cursor averaged across trials for each target for Subject A. It is clear that, after the re-positioning of the cursor based on the predicted directions, on average the subject would need to move a shorter distance to hit each target, and therefore should complete the task in less time assuming he/she would not have been distracted by the changing position of the cursor. After the cursor was placed closer to the decoded target, we simulated the cursor movement toward the target at each subject's average moving speed.

**Figure 9:**
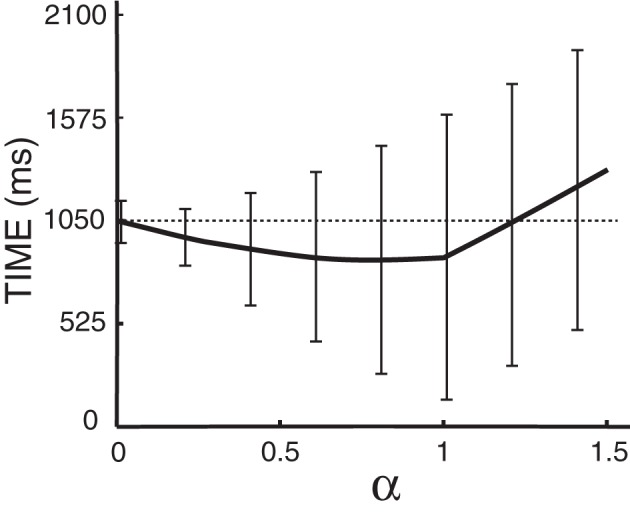
**Time to hit the target as a function of α for Subject A, where α is a scaling factor of the distance from the center to the targets**. The dashed line gives the average time to hit the target without the assistance of ECoG signals. Error bars give the standard deviation of the mean. Note that predictions from the brain signals do not have any effect when α is zero.

The distance α at which we place the cursor toward the predicted target is an important parameter that needs to be optimized. Note that α is a factor of the distance to the target (i.e., α = 0.5 places the cursor halfway between the center and the predicted target) and thus 0 < α ≤ 1. With either too small or too large of an α (α > 1), we might not take full advantage of the prediction results. The time needed to hit the target as a function α is plotted in Figure 9 for Subject A, which suggests an optimal value of 0.8 for α. The optimal α values for other subjects are listed in Table 3, which also compares the average movement time with and without the assistance of ECoG signals. The results in this table also demonstrate that the movement time of the majority of trials would have improved up to 150 ms. These encouraging results suggest that at least under certain assumptions^2^, ECoG signals may improve a user's performance in a targeting task.

**Table 3:** **Statistics and parameters of the simulated performance: optimal α values, original average movement times, average simulated movement times achieved by integrating ECoG, percentage of trials with improved movement time, percentage of trials with degraded movement time, and the statistical significance of performance improvement (in trials with reduced movement time)**.

	**Subject A**	**Subject B**	**Subject C**	**Subject D**	**Subject E**
**Optimal α**	**0.6**	**0.8**	**0.6**	**0.4**	**0.4**
Average movement time without ECoG (ms)	1050±160	1067±355	1710±156	1635±148	1507±480
Average movement time with ECoG (ms)	882±430	789±469	1604±494	1610±687	1483±733
Percentage of trials with improved movement time	61%	70%	54%	43%	53%
Percentage of trials with degraded movement time	39%	30%	46%	57%	47%
Significance (*p*-value) of improved movement time	7.3×10^−9^	1.5×10^−19^	2.7×10^−4^	6.2×10^−2^	7.4×10^−3^

## 5. Discussion

In this paper, we demonstrated that it is possible to use ECoG signals to detect the onset and the direction of an intended movement. We also demonstrate in a simulation that it may be possible to use these predictions to reduce the time to complete a targeting task by pre-positioning the cursor using ECoG signals acquired only prior to the movement. We achieved detection of movement onset by continually analyzing the incoming ECoG signals using a SVM classifier, and predicted the intended movement direction using a novel variant of a dynamic Bayesian network (i.e., the MTVDBN algorithm) that captures the spatial and temporal structure of the ECoG features.

### 5.1. Optimal integration of brain signals for improvement of performance

The distance α from the center at which the cursor is repositioned affects the improvement in performance as shown in Figure 9. Moreover, we see in Table 3 that the optimal α values ranges from 0.4 to 1.0 across subjects. It can also be observed that a higher α value is reflected in higher percentage of trials with improved movement time. This does not imply that a higher α value improves performance, which is not the case for Subjects D–E. Rather, it implies that if the directional classifier yields accurate outputs, we can take better advantage of the system (with a high α) for improving performance. Hence, improving classifier performance is crucial for the optimal integration of information from brain signals with external (e.g., joystick) control.

### 5.2. Relevance for asynchronous BCIs

Synchronous BCIs restrict the user to communicate in predefined time frames. Asynchronous BCIs, which allow the user to communicate spontaneously, may support more powerful practical applications as they are self-initiated and self-paced systems. However, asynchronous BCIs require the detection of the event in addition to identifying the properties of the event. The results presented in this paper may prove useful as the basis for an asynchronous BCI. While data collection was achieved using cued external events, our decoder was ambivalent to these events and processed the incoming data asynchronously. That is, the only input to the decoder in our experiment was the ECoG time sequence. At each time *t*, the decoder detected whether the user was beginning to move the joystick, and also predicted the intended movement. Future research could explore similar capacities in completely uncued situations, and in people who attempt but do not actually execute movements, such as people with chronic stroke.

### 5.3. Relevance for performance augmentation

We showed in a simulation that the time required to perform a directional motor task can be reduced by up to 150 ms. Such improvements in human performance should have a number of important applications. As an example, a reduction of the time to acquire a target may increase the probability to come out ahead in tactical combat. To investigate this possibility, the methods presented in this paper could be readily transferred to real-time testing. In this scenario, model parameters are estimated once initial training data are collected. The computational complexity of the MTVDBN directional classifier during the online testing session is *O*(dTM^2^), where *d* is the number of directions, *T* is the memory depth (i.e., input time samples), and *M* is the number of input features (i.e., spectral bands times the number of channels). This relatively modest computational requirement should readily support real-time testing once the MTVDBNs are trained. Nevertheless, real-time implementation and testing is necessary to determine whether the time to hit the target will also decrease in actual online experiments.

### 5.4. Experimental limitations

While the signal characteristics of ECoG are attractive, the acquisition and study of ECoG have several important limitations. Foremost, to record cortical signals subdurally, a craniotomy and dural incision must be performed. Hence, the implantation of the grids is associated with infrequent, but serious risks, such as inflammation or death. Moreover, the extent of grid coverage and its placement is not standardized across subjects and is determined by the clinical needs of the patients. For instance, the subject with the smallest fraction of trials with improved movement time (i.e., Subject D) had grids implanted ipsilateral to her dominant (left) hand. Thus, she was asked to use her non-dominant hand (i.e., contralateral to her implants) during the experiment, which might have contributed to reduced information about movement direction, and hence the low percentage of trials with improved movement times. Next, the physical and cognitive condition and level of cooperation of each subject are variable. Moreover, ECoG experiments are for practical reasons performed in uncontrolled noisy environments (i.e., hospital rooms). Furthermore, the subjects in the study suffered from epilepsy, and thus may have some degree of functional or structural reorganization compared to healthy individuals. Despite these limitations, the results presented in this and other ECoG studies are usually consistent with expectations based on human neuroanatomy.

While we controlled for important variables in this study (such as eye gaze), the experimental setup in any ECoG study is necessarily somewhat less controlled than that in the typical animal or human neuroscientific study. However, real-world environments are typically very uncontrolled as well. This circumstance strengthens our claim that our results may translate into benefits in real-life scenarios.

In its present design, the onset predictor is based on ECoG signals from the previous 1s. In other words, at least 1s of data need to be available to make a prediction about movement onset. In addition, the directional classifier is designed for prediction of discrete directions at the time of movement onset. Moreover, it takes advantage of the ±60° error margin to bring the cursor closer to the target. Hence, it is unclear to what extent the information in ECoG would generalize to reliable predictions during continuous cursor control. Finally, real-time implementation of the proposed system (i.e., re-positioning of the cursor) is required for evaluating the proposed system, as unpredicted jumps in the cursor, whether closer to or farther from the target, might affect the performance of the user.

### 5.5. Future directions

The work presented in this paper focused on detecting the onset and direction of movements using ECoG signals. The methodologies for initiation detection presented in this paper may also be extended to the detection of inhibition of a movement. Volitional inhibition is the process of adapting to sudden changes in the surrounding environment by stopping or modifying an action. Thus, future work may include testing countermanding of initiated motor responses, e.g., using a stop-signal paradigm (Logan et al., 1984). Inhibiting a response has been suggested to recruit a fronto-basal ganglia-thalamic network, including the right inferior frontal gyrus (Aron et al., 2003, 2007; Rubia et al., 2003; Chambers et al., 2006) and pre-supplementary motor cortical areas (Sumner et al., 2007; Chen et al., 2010; Scangos and Stuphorn, 2010). These areas are thought to influence the cortical areas underlying limb movement preparation and initiation, i.e., dorsal premotor (Mirabella et al., 2012) and primary motor cortices (Coxon et al., 2006; Swann et al., 2009), through the subthalamic nucleus (Aron and Poldrack, 2006; van den Wildenberg et al., 2006; Mirabella et al., 2011). Applying the methodological framework described in this study to ECoG signals collected over the right inferior gyrus, motor and pre-supplementary motor cortices during a countermanding task may thus allow for the detection of the onset of corresponding inhibitory processes. Using such an approach, it may be possible for stopping the cursor from moving in the wrong direction in cases when the directional classifier failed.

#### Conflict of interest statement

The authors declare that the research was conducted in the absence of any commercial or financial relationships that could be construed as a potential conflict of interest.

## Footnotes

^1^In ancillary analyses, we performed feature selection on 1/5th of the data, which were subsequently not used in the training and testing of the onset and direction predictors. The results of this more conservative analysis were very similar to the results reported in the paper.

^2^One assumption here is that reduced distance-to-target will reduce movement time or at least will have other distinct benefits.
